# The anabolic skeletal muscle response to acute resistance exercise is not impaired in rats fed a ketogenic diet

**DOI:** 10.1186/1550-2783-12-S1-P22

**Published:** 2015-09-21

**Authors:** C Brooks Mobley, Angelia M Holland, Wesley C Kephart, Ryan P Lowery, Petey W Mumford, Anna E McCloskey, Joshua J Shake, Paulo Mesquita, Jacob M Wilson, Michael D Roberts

**Affiliations:** 1 School of Kinesiology, Auburn University, Auburn, AL, USA; 2 Department of Health Sciences and Human Performance, The University of Tampa, Tampa, FL, USA; 3 Edward Via College of Osteopathic Medicine - Auburn Campus, Auburn, AL, USA

## Background

Many individuals that resistance train consume a typical Western diet (WD) comprised of protein, carbohydrates (many of which are sugar), and fat. Recent enthusiasm has surrounded the use of a ketogenic diet for weight loss and muscle sparing, although it is uncertain as to whether low carbohydrate diets can optimize the anabolic response to resistance training.

## Methods

This study examined the effects of KD versus WD on the anabolic response to resistance exercise using a rodent leg-kicking resistance exercise model. Male Sprague-Dawley rats (~9-10 weeks of age) were provided isocaloric amounts of either a KD (5.2 kcal/g, 20.2% protein, 10.3% carbohydrate, 69.5% fat; n = 30) or WD (4.5 kcal/g, 15.2% protein, 42.7% carbohydrate, 42.0% fat; n = 32) for 6 weeks. During week 7, the right-leg plantarflexor muscles of each rat were acutely exercised under isoflurane anesthesia using high-frequency electrical stimulations (4 sets of 8 repetitions with 2 min recovery between sets). Rats were then sacrificed at 90 min (n = 8 per group), 180 min (n = 8 per group), or 270 min (n = 8 per group) following exercise and intraperitoneal puromycin injections were provided 30 min prior to each sacrifices as a tracer for muscle protein synthesis (MPS). A subset of unexercised limbs from WD (n = 8) and KD (n = 8) were used as a non-exercise (non-EX) control comparison.

## Results

There was a main time effect for MPS, as it was significantly greater at 90, 180 and 270 min in both groups versus the non-EX condition (p < 0.001), although there was no between group effect (p = 0.59) or group*time interaction (p = 0.87). There was a main time effect for phosphorylated (p)-4E-BP1 (Thr37/46), as it was significantly greater at 90, 180 and 270 min in both groups versus the non-EX condition (p = 0.001), although there was no between group effect (p = 0.85) or group*time interaction (p = 0.93). There was a main time effect for p-rps6 (Ser235/236), as it was significantly greater at 90, 180 and 270 min in both groups versus the non-EX condition (p = 0.002), although there was no between group effect (p = 0.99) or group*time interaction (p = 0.79). There was no time effect (p = 0.31), between group effect (p = 0.42) or group*time interaction (p = 0.22) for p-AMPKα (Thr172).

## Conclusions

These data demonstrate that rats fed a ketogenic diet present a similar anabolic response to resistance exercise compared to rats fed a Western diet.

